# Reflections on China’s primary care response to COVID-19: roles, limitations and implications

**DOI:** 10.1017/S1463423622000378

**Published:** 2022-08-05

**Authors:** Xiao Tan, Chaojie Liu, Hao Wu

**Affiliations:** 1 The University of Melbourne, Melbourne, Australia; 2 La Trobe University, Melbourne, Australia; 3 Capital Medical University, Beijing, China

**Keywords:** China, COVID-19, pandemic, primary health care, public health

## Abstract

This study focuses on the role of primary care in China’s response to COVID-19. A retrospective, reflective approach was taken using data available to one of the authors who led the national community response to COVID-19, first in Wuhan and then multiple cities in ten provinces/municipalities across the country. At the peak of the pandemic, primary care providers shoulder various public health responsibilities and work in close partnerships with other key stakeholders in the local communities. Primary care providers keep playing a ‘sentinel’/surveillance role in identifying re-emerging cases after the elimination of community transmissions of COVID-19. Critically, however, the pandemic once again highlights some key limitations of the primary care sector, including the lack of gatekeeping, limited capacity and weak integration between medical care and public health.

## Introduction

**Figure 1.**
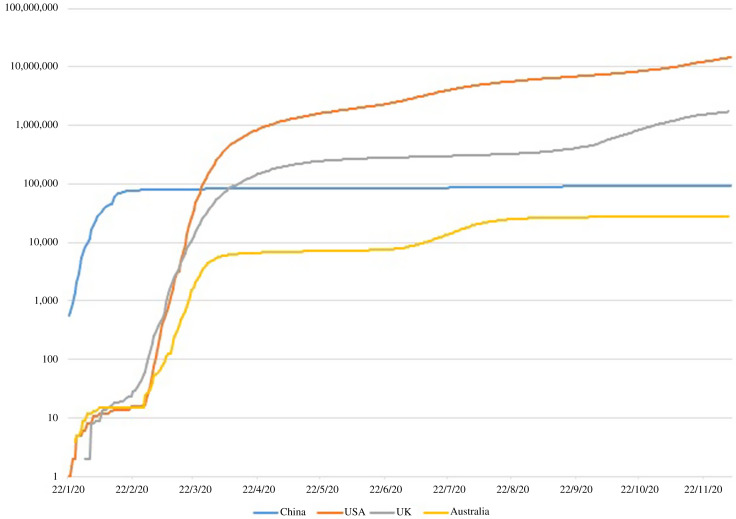
Cumulated number of confirmed COVID-19 cases (logarithmic scale), China and selected countries *Data source:* COVID-19 Data Repository by the Center for Systems Science and Engineering at John Hopkins University

In December 2019, a new type of pneumonia – later named as the coronavirus disease 2019 (COVID-19) by the WHO – was identified in Wuhan (Wang *et al.*, 2020). In the face of this unprecedented public health crisis, China rolled out strict non-pharmacological interventions, including the lockdown of the epicentre Wuhan and subsequently its surrounding cities in Hubei province since late January 2020, when the reported cumulated number of confirmed COVID-19 cases surpassed 9,000 (Zhou *et al.*, 2020). The spread of the coronavirus in China began to plateau around mid-February (Figure 1). Since late March, daily new cases had been negligible and the lockdown measures were gradually relaxed until the Omicron variant hit China.

Notably, China’s response to the pandemic is characterised by strict community management. Communities were divided into smaller geographic clusters named ‘grids’ to minimise cross-grid population movements and facilitate rapid contact tracing. Each grid was placed under close surveillance and management of designated government officials, community workers, primary care workers and volunteers (Wei *et al.*, 2020). Despite some knowledge of China’s overall approach in controlling the spread of the infections (Zhang C. *et al.*, 2020; Zhou *et al.*, 2020; Zhang P. & Gao, 2021), our understanding of the specific roles of primary care in response to COVID-19 is still limited. This study aims to fill this important gap by providing: (1) a descriptive account of how primary care responded and worked with other main stakeholders at the grassroots level during China’s fight against COVID-19; and (2) reflections on the limitations of China’s primary care and their implications in the COVID-19 context.

## Methods

A retrospective, reflective approach was taken using data available to one of the authors who led China’s national community response to COVID-19. The co-author has decades of work experience in China’s primary care settings and led the National Health Commission’s expert team for community prevention and control of COVID-19, first in Wuhan (Hubei), and later in multiple cities in ten provinces/municipalities across the country, including Beijing, Kashgar (Xinjiang), Tianjin, Chengdu (Sichuan), Shenzhen, Guangzhou, Dongguan (Guangdong), Ruili (Yunnan), Yingkou (Liaoning), Xiamen, Putian (Fujian), Nanjing, Yangzhou (Jiangsu) and Shijiazhuang (Hebei). The precious first-hand experience enabled us to discuss the topic in depth from a national perspective. In preparing for writing this paper, the co-author looked back training materials (for both primary care providers and relevant stakeholders) and other documents used during their work. It was further supplemented by data extracted from relevant policy documents and academic literature, which provided contextual information and further details to validate the findings through triangulation.

## A brief overview of China’s primary care network and functions

**Figure 2.**
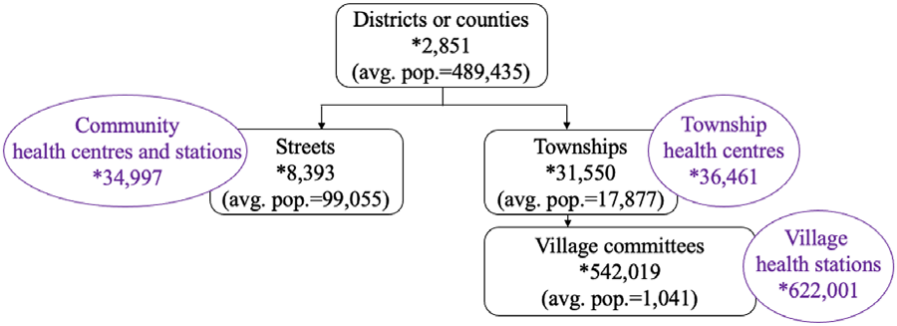
China’s primary care network *Note:* The average population given in brackets are estimated by dividing the total population by the corresponding administrative units number. We assume that the streets are urban, and townships are rural. All data in this figure are from 2018, collected from the National Bureau of Statistics, 2019).

In China, primary care services are dominated by public community health institutions: township health centres and village health stations for rural and community health centres and outreach stations for urban (Figure 2). These facilities were established through top-down planning to ensure universal coverage and avoid duplications. Each rural township has one township health centre servicing on average 20 000 populations, supplemented by one health station for each village with approximately 1,000 residents (Figure 2). Due to higher population density in urban areas, each community health centre covers a neighbourhood (administratively called ‘street’) with 30 000 to 100 000 residents (General Office of the State Council, 2020).

Compared to their counterparts in many other countries, Chinese primary care facilities have a relatively comprehensive range of functions, including essential public health services, medical care for common diseases, prescription and dispense of medicines, and rehabilitation care for certain conditions (Table 1). Reporting of and emergence response to infectious diseases also falls within the responsibility of primary care facilities, as one of the twelve categories of essential public health services (Table 1).

**Table 1.** Services provided by primary care facilities

***Essential public health services:*** Personal health records Health education Vaccinations Child health care and management Maternity care and management Aged care and management Management of chronic conditions (hypertension and diabetes) Management of severe mental disorders Management of tuberculosis Use of traditional Chinese medicines for health maintenance Reporting of and emergency response to infectious diseases Supporting health inspection activities
***Medical care:*** Diagnosis and treatment of common diseases Prescription and dispense of medicines Rehabilitation care for certain conditions

*Source:* Ministry of Finance and State Administration of Traditional Chinese Medicines (2020); General Office of the State Council (2020).

## Roles of primary care

### Key responsibilities at the peak of the pandemic

At the national level, an overall framework for primary care under the COVID-19 outbreak was set out by the government through a series of central instructions issued in January 2020 (Joint Prevention and Control Mechanism of the State Council, 2020a, 2020b; National Health Commission, 2020a, 2020b). Specifically, primary care facilities are supposed to shoulder the responsibilities including screening and triage, medical observations of quarantined people, follow-up care for hospital discharged patients, and health education (Table 2).

**Table 2.** Roles of primary care in response to COVID-19

***Screening and triage:*** Primary care workers are responsible for community-wide screening. As will be elaborated later, the large-scale screening of COVID-19 cases was performed with the help of a range of community organisations, covering all households in the communities. Primary care workers provided technical guidance to those who were involved in data collection, data analyses and identification of suspected cases. The identified suspected cases were further assessed by primary care workers and referred to designated hospitals if needed.
***Medical observations of quarantined people:*** Primary care workers are responsible for medical observations of quarantined people at home and in isolated centres. These included close contacts of confirmed cases, suspected cases, recovering patients discharged from hospitals and returned travellers from hotspots. Primary care workers took samples from the quarantined people for COVID-19 tests, carried out environmental inspections and conducted disinfection of the polluted environment.
***Follow-up care for hospital discharged patients:*** Primary care workers are responsible for the follow-up care of recovering COVID-19 patients discharged from hospitals. These included regular telephone consultations and advice to patients to comply with post-discharge follow-up tests.
***Health education:*** Primary care workers are responsible for public education in communities. Health education activities were conducted during individual consultations and through measures accessible to large populations, such as posters, pamphlets and public broadcasts.

Continuation of essential medical care is important during the outbreak of COVID-19. Although patients with fever were advised to go to designated hospitals directly, those in need of basic medical services were encouraged to visit community facilities to ease the loads of hospitals. Overall, primary care facilities experienced a significant decrease in outpatient visits: a drop of 25% between January and April 2020 compared with the same period in 2019 (National Health Commission, 2020c). On the demand side, many patients postponed their non-urgent visits out of fear of contacts with infectious patients in medical facilities. On the supply side, primary care providers were encouraged to provide repeated prescriptions for patients with well-controlled chronic conditions (Chinese Thoracic Society *et al.*, 2020; Zhang D. *et al.*, 2020). Meanwhile, telehealth consultations and health surveillance were increasingly adopted by health care providers (Liu, 2020).

COVID-19 screening became an integral part of medical care services. All patients visiting primary care facilities were subject to the screening. The patients who purchased antipyretic, antibiotics, or common cold medicines from primary care facilities or community pharmacies were registered and were later on followed up by primary care workers through phone calls. All suspected cases were referred to designated hospitals and admitted upon confirmation of the diagnosis.

In practice, the fulfilment of these functions varied across localities due to unbalanced distributions of COVID-19 cases in China (Jia *et al.*, 2020). Different implementation models were developed, which were often constrained by the community resources available (Qin *et al.*, 2021). However, there was a consensus among the primary care workers that their roles in reporting and referral of suspected cases, health education, and follow-up care for hospital discharged patients were fundamental in China’s battle against COVID-19 (Yang *et al.*, 2021).

### Working with other major stakeholders in local communities

**Figure 3.**
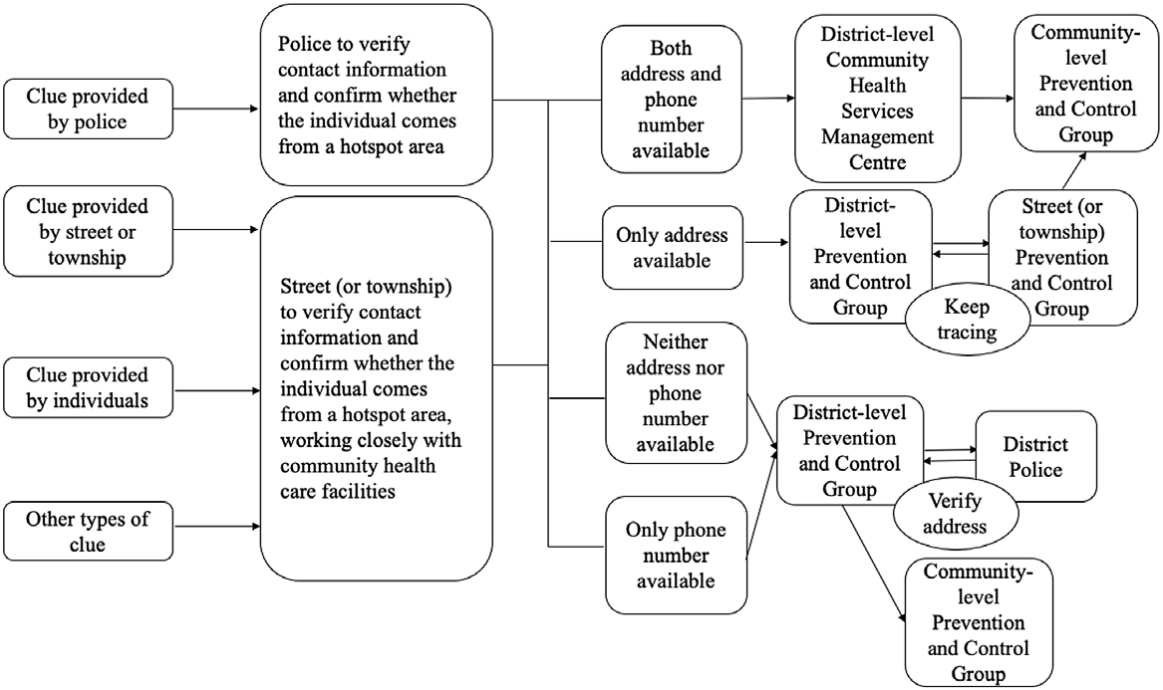
Coordination of contact tracing in Beijing *Note:* The district-level community health services management centre is responsible for supervising primary care facilities. It reports to the district-level health bureau. Prevention and control groups are set at all administrative levels, from the central level down to the county, street (or township) and community levels. They are responsible for leading and coordinating efforts in relation to the prevention and control of COVID-19.

A central leading group for COVID-19 prevention and control was established at the early stage of the COVID-19 outbreak in China as the top decision-making body. Local governments quickly followed suit by duplicating the central command structure staffed by local leaders and experts (He, Shi, & Liu, 2020). Through this command chain, central policies were followed and implemented. The local level of emergency response was determined by the local leading groups in line with the prevalence of COVID-19. The local leading groups were authorised to coordinate efforts from different governmental agencies and stakeholders to facilitate contact tracing and information sharing (Figure 3).

**Figure 4.**
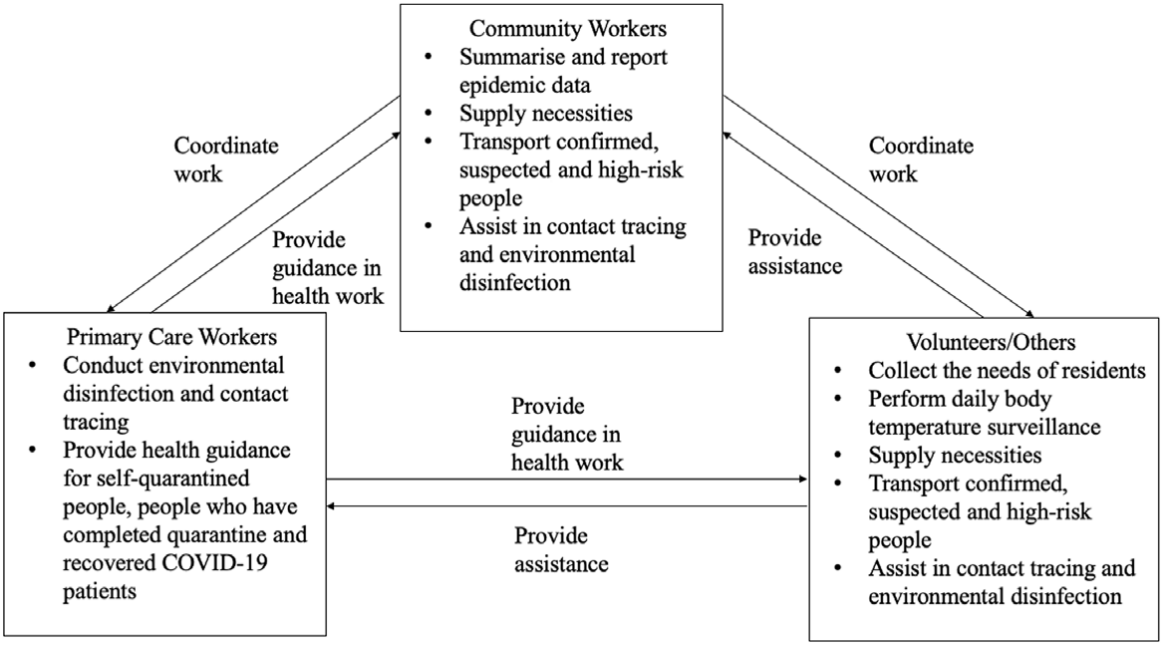
Coordination of community activities in response to COVID-19

At the grassroot level, primary care workers worked closely with other major stakeholders, whether in the community-wide screening or more targeted services for those in quarantine and isolation (Figure 4). Although the community workers and volunteers mainly focused on traffic control, transportation arrangements, and supply of necessities, they received health advice from the primary care workers in relation to these activities. They also assisted with daily body temperature surveillance, contact tracing and environmental disinfection.

### Primary care in a ‘COVID-normal’ phase

China managed to bring the spread of COVID-19 under control fairly quickly and transitioned to a ‘COVID-normal’ phase with either no community transmissions or confined sporadic outbreaks. In June 2020, the central government released guidelines for health care facilities to manage the risks in a COVID-normal phase (Joint Prevention and Control Mechanism of the State Council, 2020c). Primary care facilities are required to play a ‘sentinel’/surveillance role and take ‘first-contact responsibility’: reporting all patients with fever to the local health authority within 1 h, referring them to a fever clinic for further assessment and making follow-up calls until a formal diagnosis becomes available. In rural areas where township health centres typically provide inpatient services, designated quarantine wards are required to be set up for on-site medical observations.

## Limitations of primary care and implications

### The lack of gatekeeping, limited capacity and implications for China’s COVID-19 response

Similar to many other countries, China’s COVID-19 response was much driven by fears of overwhelming hospitals. This was particularly an issue in Wuhan because China has not established a tiered medical system – despite the comprehensive network of primary care facilities, gatekeeping is not compulsory so that patients may directly visit a specialist in tertiary hospitals without any referral (Li *et al.*, 2017). In large cities like Wuhan, many patients are used to bypassing primary care facilities and visiting tertiary hospitals for any health problem. Chinese tertiary hospitals tend to have very high volumes of outpatient visits for all kinds of diseases. Patients may experience very long waiting times for their visits. Within such a system, cross-infection can easily happen, which initially significantly exacerbated the COVID-19 infections in Wuhan. For these reasons, the key priority at the peak of the pandemic in Wuhan was to quickly establish a new tiered system in response to COVID-19. This included *fangcang* (makeshift) hospitals for mild to moderate cases and designated hospitals for serious cases. Critically, however, during the COVID-normal phase and in the longer term, the ‘sentinel’/surveillance role of primary care facilities will likely be still constrained by the lack of gatekeeping.

Relatedly, one key contributor to the lack of gatekeeping in China has been the weak capacity of primary care. Unlike in many other countries, in China, the distinction between primary care facilities and hospitals is more in capacity (in terms of space, equipment and human resources). Compared to their counterparts in hospitals, primary care doctors are typically less academically qualified, skilled and poorly paid. Establishing a general practice workforce to better separate primary care from hospitals has been a long-standing theme of China’s health care reforms, but the progress has been slow for interlocked institutional and incentive reasons. Increasing the capacity of primary care for infectious diseases thus also became a key priority for the Chinese Government, notably by building more fever clinics within primary care facilities, to separate the treatment of people with infectious diseases.

### Weak integration between medical care and public health

The pandemic also highlights that more work is needed to integrate primary care and public health. Within the Chinese primary care facilities, public health and medical services are provided by different professionals, funded by different sources of income and payment mechanisms. Despite the increase in government funding for public health services, relevant positions at primary care facilities remain unattractive in terms of salary, benefits and career prospects. During the pandemic, it became evident that there was a serious shortage of public health professionals at primary care facilities and many doctors who are routinely responsible for treating diseases have limited knowledge about infectious diseases. These issues all point to the need to further integrate medical care and public health, potentially through allocating more resources for public health, reforming the current capitation-based payments for public health services to provide better incentives, and a team-based approach to facilitate multidisciplinary collaborations. Learning experiences from other countries may also be particularly fruitful (Kinder *et al.*, 2021).

## Concluding remarks

China offers a rather unique experience in its response to COVID-19, characterised by strict community management to pursue a zero-COVID strategy. The surge in workforce capacity was enabled by the Chinese Government mobilising a range of stakeholders to work collaboratively. Primary care was an integral part of this massive community response and provided various functions. China’s model proved effective in quickly controlling the spread of COVID-19, but some key weaknesses of primary care have been once again exposed, notably the lack of gatekeeping, limited capacity and weak integration between medical care and public health. The applicability of China’s model to other countries may be limited due to significant differences in socio-political, economic and health care system contexts. However, within the country, case studies of where the model worked well or less well would have great educational value and would be a productive next step in considering the Chinese experience in more detail.

## References

[ref1] Chinese Thoracic Society, Chinese Society of General Practice, Chinese Association of Chest Physician, Chinese Medical Doctor Association General Practitioners Sub-Association, Chinese Socitey of Infectious Disease, Chinese Alliance for Respiratory Diseases in Primary Care, and Expert Group of Expert Recommendations for the Prevention and Control of Novel Coronavirus Infections in Primary Care (2020) Expert recommendations for the prevention and control of COVID-19 virus infection in primary care (first edition). China Journal of General Practitioners 19, 175–192.

[ref2] General Office of the State Council (2020) *Outline for the Planning of the National Medical and Health Service System*. Retrieved 25 November 2020 from http://www.gov.cn/zhengce/content/2015-03/30/content_9560.htm.

[ref3] He AJ , Shi Y and Liu H (2020) Crisis governance, Chinese style: distinctive features of China’s response to the Covid-19 pandemic. Policy Design and Practice 3, 242–258. DOI: 10.1080/25741292.2020.1799911

[ref4] Jia JS , Lu X , Yuan Y , Xu G , Jia J and Christakis NA (2020) Population flow drives spatio-temporal distribution of COVID-19 in China. Nature 582, 389–394. DOI: 10.1038/s41586-020-2284-y 32349120

[ref5] Joint Prevention and Control Mechanism of the State Council (2020a) *Community Work Plan for the Prevention and Control of COVID-19 (Trial)*. Retrieved 25 November 2020 from http://www.nhc.gov.cn/jkj/s3577/202001/dd1e502534004a8d88b6a10f329a3369.shtml.

[ref6] Joint Prevention and Control Mechanism of the State Council (2020b) *Notice on Further Improving the Prevention and Control of COVID-19 in Rural Areas*. Retrieved 25 November 2020 from http://www.gov.cn/xinwen/2020-02/27/content_5483966.htm.

[ref7] Joint Prevention and Control Mechanism of the State Council (2020c) *Notice on Health Care Facilities Playing a Sentinel Role in the Normalised Work of Preventing and Controlling COVID-19*. Retrieved 25 November 2020 from http://www.gov.cn/xinwen/2020-06/11/content_5518727.htm.

[ref8] Kinder K , Bazemore A , Taylor M , Mannie C , Strydom S , George J and Goodyear-Smith F (2021) Integrating primary care and public health to enhance response to a pandemic. Primary Health Care Research & Development 22, 1–7. DOI: 10.1017/S1463423621000311 PMC8220344 34109936

[ref9] Li X , Lu J , Hu S , Cheng KK , de Maeseneer J , Meng Q , Mossialos E , Xu DR , Yip W , Zhang H , Krumholz HM , Jiang L and Hu S (2017) The primary health-care system in China. The Lancet 380, 2584–2594. DOI: 10.1016/S0140-6736(17)33109-4 29231837

[ref10] Liu C (2020) Health information systems amid COVID-19 outbreak: lessons from China. Advanced online publication. Health Information Management Journal. DOI: 10.1177/1833358320947557 32806959

[ref11] Ministry of Finance and State Administration of Traditional Chinese Medicines (2020) *Notice on Effectively Carrying Out the Work of Providing National Essential Public Health Services*. Retrieved 25 November 2020 from http://www.nhc.gov.cn/jws/s7874/202006/619506aa0fd14721b7e5711d389c323f.shtml.

[ref12] National Bureau of Statistics (2019) China Statistical Yearbook 2019. Beijing: China Statistics Press.

[ref13] National Health Commission (2020a) *Notice on Strengthening Primary Care Facilities in the Prevention and Control of COVID-19*. Retrieved 25 November 2020 from http://www.gov.cn/zhengce/zhengceku/2020-01/27/content_5472401.htm.

[ref14] National Health Commission (2020b) *Notice on Further Improving the Prevention and Control of COVID-19 in Primary Care Settings*. Retrieved 25 November 2020 from https://www.chinagp.net/Home/Content/show/id/4839.do.

[ref15] National Health Commission (2020c) *Current Situation of National Medical Services, January*–*April 2020*. Retrieved 25 November 2020 from http://www.nhc.gov.cn/mohwsbwstjxxzx/s7967/202007/c8c897a6320d47c59c8f950f8acd2a23.shtml.

[ref16] Qin Y , Huang Y , He Z , Zhang J , Luo L and Tang G (2021) Prevention and control of major infectious diseases in primary health care organizations: experiences, problems and countermeasures from case-based studies, Chinese General Practice 24, 11–16.

[ref17] Wang C , Horby PW , Hayden FG and Cao GF (2020) A novel coronavirus outbreak of global health concern, Lancet 395, 470–473. DOI: 10.1016/S0140-6736(20)30185-9 31986257 PMC7135038

[ref18] Wei Y , Ye Z , Cui M and Wei X (2020) COVID-19 prevention and control in China: Grid governance. Journal of Public Health, fdaa175. DOI: 10.1093/pubmed/fdaa175 PMC7543388 32978620

[ref19] Yang C , Li Y , Li Z , Yang H , Ni Y , Lin J , Wang D , Xu H and Song H (2021) Roles of primary care doctors in COVID-19 pandemic: level of consistency across perceptions of doctors and experts and impact factors. Chinese General Practice 24, 17–22.

[ref20] Zhang C , Cao GF , Wang Z and Wu H (2020) Fighting against COVID-19 at the community level – Wuhan City, Hubei Province, China, 2020. China CDC Weekly 2, 463–466. 34594680 10.46234/ccdcw2020.126 PMC8393059

[ref21] Zhang D , Yao M , Wang J , Ye D , Chen Q , Guo F , Zou C , Lin K , Lu H , Huang X , Zheng J , Chi C and Zhong N (2020) Guidance on the control and prevention of SARS-CoV-2 infection in primary healthcare institutions in rural China (first edition). Chinese General Practice 23, 763–769.

[ref22] Zhang P and Gao J (2021) Evaluation of China’s public health system response to COVID-19. Journal of Global Health 11, 05004. DOI: 10.7189/jogh.11.05004 33643637 PMC7897426

[ref23] Zhou L , Wu Z , Li Z , Zhang Y , McGoogan JM , Li Q , Dong X , Ren R , Feng L , Qi X , Xi J , Cui Y , Tan W , Shi G , Wu G , Xu W , Wang X , Ma J , Suu X , Feng Z , Gao GF and China COVID-19 Task Force (2020) One hundred days of coronavirus disease 2019 prevention and control in China. Clinical Infectious Diseases, ciaa725. DOI: 10.1093/cid/ciaa725 PMC7314211 33501949

